# Objections to Cold Applications to the Extremities in Gout; with a Case

**Published:** 1815-09

**Authors:** 


*For the London Medical and Physical Journal.*

### *Objections to Cold Applications to the Extremities in Gout; with a Case.* By N. W.

I BELIEVE the majority of Dr. Kinglake's professional brethren agree with him in preferring the cooling treatment of gout to any other method that has hitherto been adopted; but at the same time I verily believe there is not a solitary individual amongst them, but as thoroughly disagrees with him as to the expediency of using cold topical applications to the extent he is or has been wont to advise them.

I apprehend it is an undeniable fact, that, where so great a degree of increased arterial action prevails, as is invariably the case at the articulations of the extremities when under the influence of asthritic excitement, a sympathetic disposition, at least, to increased vascular action in a smaller degree pervades the whole sanguiferous system.

That position then admitted, I take leave to suggest whether, by repelling such an high degree of inflammatory action from the joint of an extremity (where it might remain perfectly harmless as far as the immediate dissolution of life would be involved in its effects), a considerable risk might not be incurred of its invading some other part more immediately connected with vital preservation, unless steps were taken before-hand for diminishing the quantity of circulating fluid, and for abating its general impetus.

My opinion is, that, when such a practice is adopted without having recourse to these precautionary measures, the risk of of destroying life is always present, as the following case will, I think, go a great way to confirm.

On Saturday night, the 9th of April, 1814, I was called to J. C. who lived in the country at a small distance from this town, a tall, spare, robust man, of temperate habits, and about forty years of age. I found him delirious, his face and eyes extremely red, the vessels of the temples and neck throbing with uncommon violence; in short, labouring under all the symptoms of phrenitis in their most aggravated form.

Knowing that he had for some days before been enduring an inflammatory rheumatic affection of one of his knees, and that on previous similar occasions he had used cold application, after the manner recommended by Dr. Kinglake, I suspected his present phrenitic attack might have been produced by the translation of the morbid affection of the knee to the head, and enquiry proved the correctness of my suspicion. I was told that, although he had been particularly requested not to do so, he had been applying cloths made wet with cold water to his knee, and replacing them with others so often as heat or pain indicated the change, and that about an hour before my arrival he had exclaimed, "I am a dead man—I shall die—the pain has left my knee and struck to my head!" Every appearance of inflammation had vanished from the knee. From eighteen to twenty ounces of blood were immediately drawn through a large orifice from the arm; cold water, by means of cloths, were applied to the head after the manner they had heretofore been applied to the knee, and both knees were directed to be enveloped with sinapisms. As speedily as it could be procured, a brisk purge was given and a large blister applied between the shoulders. The next morning early, no abatement of symptoms having occurred, ten or twelve ounces of blood were taken from the temporal artery; the head shaved and covered with a blistering plaster; and, I believe, every other means were suggested that could in any wise tend to avert the impending danger—but all in vain; he died on the Monday morning, about thirty-six hours from the commencement of the phrenitic attack.

*Langport;* N. W.
*July* 18, 1815.

